# Dysregulation of Translation in TDP-43 Proteinopathies: Deficits in the RNA Supply Chain and Local Protein Production

**DOI:** 10.3389/fnins.2022.840357

**Published:** 2022-03-07

**Authors:** Reed T. Bjork, Nicholas P. Mortimore, Suvithanandhini Loganathan, Daniela C. Zarnescu

**Affiliations:** ^1^Department of Molecular and Cellular Biology, University of Arizona, Tucson, AZ, United States; ^2^Neuroscience Graduate Interdisciplinary Program, University of Arizona, Tucson, AZ, United States

**Keywords:** TDP-43, ALS, FTD, translation, axon, dendrite, synapse, neurodegeneration

## Abstract

Local control of gene expression provides critical mechanisms for regulating development, maintenance and plasticity in the nervous system. Among the strategies known to govern gene expression locally, mRNA transport and translation have emerged as essential for a neuron’s ability to navigate developmental cues, and to establish, strengthen and remove synaptic connections throughout lifespan. Substantiating the role of RNA processing in the nervous system, several RNA binding proteins have been implicated in both developmental and age dependent neurodegenerative disorders. Of these, TDP-43 is an RNA binding protein that has emerged as a common denominator in amyotrophic lateral sclerosis (ALS), frontotemporal dementia (FTD) and related disorders due to the identification of causative mutations altering its function and its accumulation in cytoplasmic aggregates observed in a significant fraction of ALS/FTD cases, regardless of etiology. TDP-43 is involved in multiple aspects of RNA processing including splicing, transport and translation. Given that one of the early events in disease pathogenesis is mislocalization from the nucleus to the cytoplasm, several studies have focused on elucidating the pathogenic role of TDP-43 in cytoplasmic translation. Here we review recent findings describing TDP-43 translational targets and potential mechanisms of translation dysregulation in TDP-43 proteinopathies across multiple experimental models including cultured cells, flies, mice and patient derived neurons.

## Introduction

One of the most unique features of a neuron is its dramatic morphology. Aside from the requisite cell body, which contains the nucleus and as little as 1% of the cytoplasm, neurons typically wield a robust network of processes including dendrites and a single axon (reviewed in Holt et al., 2019). In humans, the axon can extend beyond a meter in length and forge tens of thousands of synaptic connections (reviewed in Holt et al., 2019). Dendrites are highly branched processes and serve as the postsynaptic component of the synapse. This level of compartmentalization poses unique challenges and opportunities for spatiotemporally sensitive processes, especially for critical neuronal functions such as neural and synaptic plasticity. More specifically, neurite outgrowth, maintenance, branching, axonal turning, synaptogenesis, and synapse maintenance require carefully orchestrated, spatially and temporally controlled protein synthesis, which is achieved in part through mRNA localization and local translation (reviewed in Sutton and Schuman, 2006; Batista and Hengst, 2016; Costa and Willis, 2018; Holt et al., 2019). A growing body of work over the last two decades has revealed significant dysregulation of translation in several neurodevelopmental and neurodegenerative diseases such as Fragile X Syndrome (FXS), autism spectrum disorders (ASD), amyotrophic lateral sclerosis (ALS), spinal muscular atrophy (SMA), and Alzheimer’s Disease (AD) (reviewed in Bear et al., 2008; Akins et al., 2009; Liu-Yesucevitz et al., 2011; Baleriola et al., 2014; Ferro et al., 2018). Therefore, local translation has emerged as a critical mechanism underlying the pathogenesis of neurological disorders across lifespan.

Regulated primarily by RNA binding proteins, proper maintenance of RNA homeostasis is necessary for healthy function of the neuron. Aberrant function of several RNA binding proteins including TAR DNA-binding protein 43 (TDP-43), Fused in Sarcoma (FUS) and Senataxin (SETX) has been linked to neurodegenerative disease pathogenesis. TDP-43 is of particular interest as it participates in numerous RNA processing steps and has been identified as a major component of pathological cytoplasmic inclusions in ALS and frontotemporal dementia (FTD) (Andersen and Al-Chalabi, 2011). These cytoplasmic accumulations, collectively referred to as TDP-43 proteinopathy, are a hallmark of neuron degeneration in multiple disorders including ALS (97% of cases), FTD (45% of cases) (Ling et al., 2013), Alzheimer’s disease (AD, 57% of cases), and Lewy Body Dementia (McAleese et al., 2017). Together with the identification of disease causative mutations in 2–4% of ALS patients, these findings establish TDP-43 as a common denominator across multiple neurodegenerative diseases.

Recent reports have identified TDP-43 dependent alterations in the translatome (MacNair et al., 2016; Neelagandan et al., 2019; Marques et al., 2020; Lehmkuhl et al., 2021). Whether this occurs directly, via TDP-43 association with the translation machinery or indirectly, by disturbing cellular homeostasis, recently reported translation targets of TDP-43 proteinopathy have uncovered a plethora of cellular pathways that are providing insights into potential therapeutic strategies for ALS. Here we highlight recent discoveries and mechanistic insights into TDP-43 dependent translation dysregulation in ALS/FTD.

## Translation Dysregulation in TDP-43 Proteinopathy

### TDP-43 Structural Features—Implications for Translation

**FIGURE 1:**
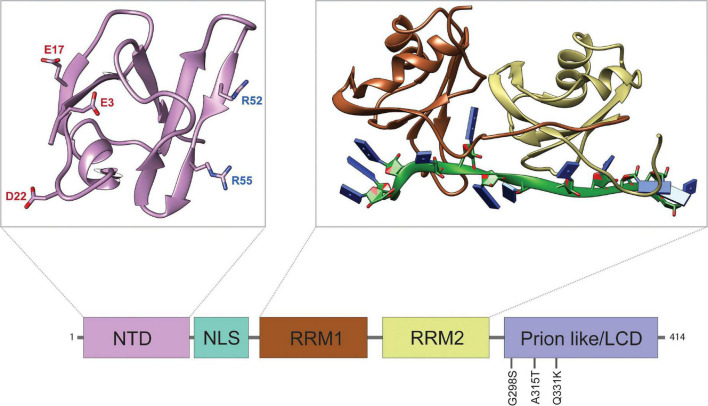
Structure and Domain Features of TDP-43. Domain map of TDP-43 depicting the approximate sizes of functional domains. The disease-associated mutations discussed in this review are highlighted. From left to right: N-terminal domain (NTD), nuclear localization signal (NLS), RNA-recognition motif 1 (RRM1), RNA-recognition motif 2 (RRM2), and low complexity domain (LCD). Top left panel: structure of the NTD (PDB ID: 5MDI) with aggregation antagonizing amino acid residues E3, E17, D22, R52, and R55, as indicated (Afroz et al., 2017). The negatively charged NTD residues (red) form salt bridges with positively charged residues (blue) of other TDP-43 NTDs to form oligomers. Top right panel: structure of the tandem RRMs bound to UG-rich RNA (PDB ID: 4BS2) (Lukavsky et al., 2013). The RNA-structure is shown in green with nucleobases represented by blue rectangles.

TDP-43 is an evolutionarily conserved DNA/RNA binding protein comprising four primary domains: an N-terminal domain (NTD, aa: 1–103) with a nuclear localization signal (NLS, aa: 82–98), two RNA-recognition motifs (RRMs, aa: 104–200, 191–262), and an intrinsically disordered C-terminal low-complexity domain (LCD, aa: 274–413) (see Figure 1). Insights from key structural features and disease causative mutations in TDP-43 highlight important mechanisms that drive TDP-43 pathogenesis. The vast majority of ALS/FTD causing mutations reside within its LCD and were shown to increase the propensity of TDP-43 to aggregate (Johnson et al., 2009; Buratti, 2015). In cases of familial ALS the A315 residue is mutated to E or T (A315E and A315T), which can lead to the assembly of tightly packing steric zippers that form stable fibrils (Nelson et al., 2005). The A315E mutation and phosphorylation of A315T also introduce electrostatic interactions into the amyloidogenic core regions of TDP-43, stabilizing larger fibril conformations that can seed irreversible aggregates (Cao et al., 2019). These TDP-43 aggregates could cause translation inhibition of sequestered mRNAs or affect translation by association with factors that regulate protein synthesis (Ramaswami et al., 2013). Interestingly, oligomerization of TDP-43 through the N-terminal domain via salt bridges between D22 and R55, E3 and R52, as well as E17 and R52 has been shown to antagonize TDP-43 proteinopathy by spatially separating LCDs that may otherwise aggregate (Afroz et al., 2017). Furthermore, the tandem RRMs of TDP-43 are of central importance to the specificity and activity of the wild-type protein, contributing to most of its RNA binding activity. The two RRMs have distinct nucleotide specificities, but mainly recognize (UG/TG)n repeat RNA via the organization of key aromatic and charged residues (Lukavsky et al., 2013). Recent findings suggest that the RNA binding activity of TDP-43 may be of particular interest in disease, as association with long sequences of UG rich RNA has been shown to dissolve optogenetically induced TDP-43 aggregates *in vitro* and reduce neurotoxicity (Mann et al., 2019; Grese et al., 2021). Another interesting feature of the RRM domains is the presence of a salt bridge, R151/D247, that is not required for RNA binding *per se*, but regulates affinity and specificity (Flores et al., 2019). Disrupting the R151/D247 salt bridge affects RNA binding and destabilizes the protein, which mitigates toxicity.

Recently, a short TDP-43 (sTDP-43) isoform upregulated by neuronal hyperactivity has been identified (Weskamp et al., 2020). It results from alternative splicing that removes most of the C terminus unstructured domain and introduces a functional nuclear export signal (NES) that causes it to localize to the cytoplasm where it can drive the aggregation of endogenous, full length TDP-43 (Weskamp et al., 2020). TDP-43 has also been shown to undergo liquid-liquid phase separation (LLPS) via its LCD, which may facilitate aggregation (Babinchak et al., 2019; Conicella et al., 2020; Pakravan et al., 2021). A recent study characterized a novel mouse model expressing LLPS-deficient TDP-43 (TDPΔCR) caused by deletion of residues 321–340 that represent the conserved region (CR) required for LLPS from within the LCD (Gao et al., 2021). Structure function studies defined residues 99–105 as the TDP-43 translation factor binding motif due to its ability to bind several translation factors and its requirement for enhanced protein synthesis observed in the TDPΔCR mouse model (Gao et al., 2021). Altogether, these findings highlight the importance of RNA binding, RNA sequestration as well as TDP-43 protein aggregation in disease pathology, and outline potential avenues for TDP-43 involvement in translational control.

### TDP-43 Mislocalization to the Cytoplasm Is a Critical Event in Disease Pathogenesis

Under physiological conditions, TDP-43 resides predominantly in the nucleus where it modulates transcription, splicing and miRNA biogenesis (Casafont et al., 2009). Due to its scarce presence outside the nucleus, the extent to which full length TDP-43 performs physiological operations in the cytoplasm remains unclear, considering that the predicted NES does not function as such (Ederle et al., 2018). In numerous neurodegenerative diseases, however, TDP-43 is depleted from the nucleus and mislocalizes to the cytoplasm forming pathological inclusions (Neumann et al., 2006; McAleese et al., 2017). Substantiated by these observations, the mechanism of TDP-43 pathogenesis is postulated as a loss of nuclear function and/or cytoplasmic gain-of-function (Vanden Broeck et al., 2014; Ederle and Dormann, 2017). To test the nuclear depletion hypothesis, early studies using gene knockout methods revealed that loss of TDP-43 is lethal, both in embryonic development and postnatally when inactivated using Cre-inducible gene excision (Kraemer et al., 2010; Sephton et al., 2010; Wu et al., 2012). Other studies focused their attention on recapitulating the cytoplasmic accumulation of TDP-43, demonstrating that overexpression of either the full length wild type protein (Johnson et al., 2008; Li et al., 2010; Liachko et al., 2010; Tsai et al., 2010; Wils et al., 2010; Igaz et al., 2011) or a disease-associated variant (Kabashi et al., 2009; Wegorzewska et al., 2009; Liachko et al., 2010; Zhou et al., 2010; Estes et al., 2011; Huang et al., 2012), is sufficient to cause ALS phenotypes in various animal models. Using other mutant forms of TDP-43 to manipulate its localization and function provided additional support for cytoplasmic toxicity as a likely disease mechanism. For instance, variants of TDP-43 harboring RRM mutations that affect RNA binding do not result in cytoplasmic mislocalization or induce toxicity (Elden et al., 2010; Voigt et al., 2010; Ihara et al., 2013; Coyne et al., 2015). Conversely, deletion of the NLS causes TDP-43 (ΔNLS-TDP-43) to accumulate in the cytoplasm inducing toxicity in primary cortical neurons of rats and neurodegeneration in mice, with rare cytoplasmic inclusions in motor neurons (Barmada et al., 2010; Igaz et al., 2011; Walker et al., 2015; Spiller et al., 2016). Collectively, these animal models support both loss and gain of function mechanisms and highlight the relationship between cytoplasmic accumulation of TDP-43 and neurodegeneration.

### TDP-43, Stress Granules, and Translation

As an RNA binding protein, TDP-43 has a broad influence on gene expression, however, its impact on translation in particular is not well understood. Aside from direct alterations to the translation machinery, disturbances in granule dynamics and mRNA localization may contribute considerably to changes in the translatome. As new transcripts are generated in canonical cap-dependent translation (reviewed in Jackson et al., 2010), the growing pre-mRNA strands are available to associate with RNA binding proteins and form highly dynamic ribonucleoprotein (RNP) complexes (Moore and Proudfoot, 2009). The composition of an RNP complex changes with respect to its location and function in RNA metabolism, providing a mechanism for controlling the translational fate of the mRNAs it associates with (Singh et al., 2015). In the cytoplasm, translationally repressed RNP complexes can remodel into higher order structures such as stress granules, neuronal RNA transport granules, and P-bodies (Bowden and Dormann, 2016). Stress granules are a well-studied subset of RNP complexes that can rapidly assemble in response to stress or when translation initiation is inhibited, resulting in sequestration of mRNAs and translation initiation factors (reviewed in Anderson and Kedersha, 2009; Buchan and Parker, 2009). The abundance of highly disordered, prion-like domains in several RNA binding proteins predisposes stress granules to pathogenicity (King et al., 2012; Ramaswami et al., 2013). As proposed in the ribostasis hypothesis, stochastic assembly of RNA binding proteins within stress granules into self-propagating amyloid structures creates aberrant RNP aggregates that disrupt RNA homeostasis eventually leading to cell death (Ramaswami et al., 2013). Disease associated TDP-43 variants (A315T, M337V, Q343R, R361S) have been shown to disrupt stress granule dynamics (McDonald et al., 2011; Liu-Yesucevitz et al., 2014; Gordon et al., 2019). Although recent reports do not support the involvement of stress granules in TDP-43 proteinopathy, or suggest that TDP-43 association with stress granules may in fact be protective (McGurk et al., 2018; Gasset-Rosa et al., 2019; Mann et al., 2019), loss of function studies for the stress granule assembly factor G3BP1 revealed that stress granules facilitate but are not required for TDP-43 aggregation (Fernandes et al., 2020).

Translation can also occur independent of the 5′ cap, driven by the presence of Internal Ribosome Entry Sites (IRESs), initially discovered within viral RNAs and subsequently identified within mRNAs with certain structural features (Pelletier and Sonenberg, 1988). Cap independent translation mechanisms remain less understood compared to canonical translation and rely on RNA binding proteins known as IRES Trans-Activating Factors (ITAFs), which also play a variety of other roles in the cell (Hellen and Sarnow, 2001; Komar and Hatzoglou, 2011). Cap independent translation is prevalent during cellular stress and may play a role in neurological disorders such as ALS/FTD where stress granule dynamics appears to be altered (reviewed in Pelletier and Sonenberg, 1988; Hellen and Sarnow, 2001; Spriggs et al., 2008; Komar and Hatzoglou, 2011; Yang and Wang, 2019).

### TDP-43—Ribosome Association and Effects on Translation

The ribostasis hypothesis posits that cytoplasmic accumulation of TDP-43 leads to mRNA sequestration and translation inhibition. In support of this hypothesis, various reports indicate that TDP-43 may act as a global repressor of translation. Indeed, TDP-43 depletion in HEK cells by siRNA causes a global increase in translational yield (Freibaum et al., 2010; Fiesel et al., 2012). Corroborating these findings, recent studies show that overexpression of cytoplasmically restricted TDP-43 (ΔNLS-TDP-43) in HEK or SHSY5Y cells causes a global reduction in protein synthesis, as demonstrated by Surface Sensing of Translation (SUnSET) assays that quantify puromycilation of nascent proteins (Russo et al., 2017; Charif et al., 2020). Further substantiating these findings *in vivo*, overexpression of cytoplasmic TDP-43 in the forebrains of transgenic mice (ΔNLS-TDP-43) results in decreased global translation as evidenced by both SUnSET and polysome profiling (Charif et al., 2020). Interestingly, puromycin incorporation experiments in TDPΔCR mice and primary neurons revealed an increase in global protein synthesis. Since ribosomal protein levels were unaltered, the authors concluded that ribosome assembly was increased (Gao et al., 2021).

TDP-43-induced changes in translation may occur through TDP-43 association with components of the protein synthesis machinery (Freibaum et al., 2010). For instance, induction of short-term oxidative stress in HeLa cells causes TDP-43 to shift from non-ribosomal fractions to monosomal fractions where it associates with stalled ribosomes via mRNA binding (Higashi et al., 2013). Following longer recovery time after arsenite induced stress, TDP-43 associates temporarily with polyribosomes indicating its ability to act both as a negative and as a positive regulator of translation (Higashi et al., 2013). Subsequent reports that TDP-43 co-migrates with non-ribosomal fractions and polyribosomes in Drosophila models of TDP-43 proteinopathy lend support to the notion that TDP-43 also associates with the translation machinery *in vivo* and may play a pivotal role in the dysregulation of translation in disease (Coyne et al., 2015). Consistent with these findings, polysome profiling in a motor neuron-like cell line or primary cortical neurons transfected with human TDP-43 (hTDP-43) shows that in the absence of stress, TDP-43 fractionates with polyribosomes and shifts to lighter RNP fractions upon polysome disruption with EDTA treatment (Neelagandan et al., 2019). Further analyses using ribosome profiling confirmed that TDP-43 associates with polyribosomes in actively translating cells (Neelagandan et al., 2019). Furthermore, ribosome footprinting in primary cortical neurons identified specific mRNA translational targets of TDP-43 (Neelagandan et al., 2019; see more in “Specific Candidate Targets,” below and in Table 1).

**TABLE 1:** Summary of key findings on the role and mechanism of TDP-43 in regulating translation.

Manuscript	Model	TDP-43 variant/expression	Key methods	Key findings	Effects on translation	Targets/Mechanism
Altman et al. (2021)	Mice; primary neurons; patient-derived stem cell MNs	ΔNLS-TDP-43	Microfluidic culture; dox TET-off; IF; RNA IP; OPP incorporation	Cytoplasmic TDP-43 forms axonal RNP condensates, reduces local protein synthesis. Restoring TDP-43 localization reinnervates NMJs.	Axon; NMJ	Mitochondrial proteins (ATP5A1, Cox4i1, Ndufa4)
Gao et al. (2021)	Mice; HEK293 cells	TDPΔCR	Behavior; IF; IHC; SUnSET; electrophys.	Behavior/neuronal abnormalities without TDP-43 proteinopathy. Global increase in protein synthesis. Enrichment of specific targets.	Global	PABPC4, PABPC1, RPS6, EEF1A1, RPL7
Lehmkuhl et al. (2021)	Drosophila MNs; patient spinal cords	TDP-43^WT^; TDP-43^G298S^ (overexpressed); TBPH^RNAi^	TRAP; RNA IP; RNA seq. Bioinformatics	Identified novel target. TDP-43 proteinopathy causes loss of Dlp at NMJ, increase/dlp puncta at neuropil.	Global; NMJ	*Dlp*
Wong et al. (2021)	Mice; primary neurons; HEK293 cells	Endogenous; TDP-43 Tg (overexpressed)	dSTORM; IF; RPM;	TDP-43 associates with FMRP, Staufen on RNP granules. TDP-43 proteinopathy prevents activation-induced dissolution of RNPs.	Dendrites	*Map1b*, GluR1, *CamKII*
Nagano et al. (2020)	Cortical neurons	TDP-43 knockdown (shRNA)	MS2 tagging system; FISH; IF; IHC; RNA IP	TDP-43 is necessary for transport of ribosomal protein mRNA in axons.	Axons	Ribosomal proteins (Rp141, Rp126, Rps7)
Marques et al. (2020)	Mice	TDP-43^A315T^; hTDP-43 hemizygous control	TRAP; RNA seq.	Identified novel translational targets of TDP-43 associated with onset of motor symptoms.	Global	Syngr4 (up), Plekhb1 (down)
Charif et al. (2020)	Mice; HEK293 cells	ΔNLS-TDP-43 (overexpressed)	SUnSET; Polysome profiling; IHC	Global reduction in protein synthesis, *in vitro* and *in vivo*.	Global	
Chu et al. (2019)	Primary hippocampal neurons; pyramidal neurons	Endogenous; TDP-43 knockdown (siRNA)	TRICK RNA biosensor; RNA FISH/IF	TDP-43 cooperates with FMRP, Staufen1 to regulate dendritic mRNA transport.	Dendrites	*Rac1*
Neelagandan et al. (2019)	MN like cells; primary cortical neurons	hTDP-43; TDP-43^A315T^ (overexpressed)	TRAP; ribosome footprinting; polysome profiling	TDP-43 associates with ribosomes. Enhanced translation of specific targets.	Global	*Camta1*, *Mig12*, *Dennd4a* (A315T)
Russo et al. (2017)	SHSY5Y neuroblastoma cells	ΔNLS-TDP-43 (overexpressed)	SUnSET; polysome profiling; IF	TDP-43 associates with ribosomes via RACK1. Global reduction in protein synthesis.	Global	RACK1
Coyne et al. (2017)	Drosophila MNs	TDP-43^WT^; TDP-43^G298S^ (overexpressed)	Polysome profiling; IHC	TDP-43 impairs hsc70-4 translation by mRNA sequestration.	NMJ	*hsc70-4*
Ishiguro et al. (2016)	*Escherichia coli*; HEK293 cells	Endogenous, purified TDP-43	SELEX	TDP-43 associates with G quadruplex containing mRNAs.	Global	G quadruplex mRNAs
MacNair et al. (2016)	Mice; spinal cord motor neurons	TDP-43^A315T^ (overexpressed)	TRAP; microarray analysis; IF; IHC	Identified two novel translational mRNA targets of TDP-43.	Global	DDX58 (up), MTHFSD (down)
Majumder et al. (2016)	HEK293 cells; primary mouse hippocampal neurons;	TDP-43 knockdown (RNAi)	FISH; RNA IP; IF	TDP-43 and FMRP co-repress translation of specific mRNAs.	Global; dendrites	*Rac1*, *Map1b*, *GluR1*

*MN, motor neuron; NMJ, neuromuscular junction; IF, immunofluorescence; SUnSET, surface sensing of translation; IP, Immunoprecipitation; IHC, immunohistochemistry; RPM, ribopuromycylation; dSTORM, direct stochastic optical reconstruction microscopy; OPP, O-propargyl-puromycin; FISH, fluorescence in situ hybridization; SELEX, systematic evolution of ligands by exponential enrichment.*

These studies suggest that TDP-43 may play a direct role in translational control, perhaps by direct association with ribosomes. In SHSY5Y neuroblastoma cells, TDP-43 associates with ribosomes via RACK1, a WD40 scaffold protein that binds the 40S ribosomal subunit near the mRNA exit channel and functions as a docking site for several translation machinery proteins (Gallo and Manfrini, 2015; Russo et al., 2017). Immunostaining of mouse hippocampal neurons indicates that TDP-43 puncta strongly colocalize with RACK1 containing granules that correspond to ribosomes/polyribosomes within neurites. Furthermore, RACK1 colocalizes with TDP-43 in pathological inclusions in ALS patient spinal cords, further substantiating the notion that TDP-43’s association with ribosomes may promote TDP-43 aggregation and neurodegeneration (Russo et al., 2017). Several reports highlight TDP-43 dependent alterations in translation that may be indirect, resulting from impaired cellular homeostasis. When used in mouse or Drosophila models of TDP-43 proteinopathy, Translating Ribosomes Affinity Purification (TRAP) revealed complex changes in several targets and pathways within the *in vivo* motor neuron translatome, including RNA metabolism and cytoplasmic translation itself (MacNair et al., 2016; Lehmkuhl et al., 2021). Additionally, TDP-43 overexpression in Drosophila has been shown to cause the phosphorylation of eukaryotic initiation factor-2alpha (eIF2alpha), an indicator of stress granule formation and consequently, translational repression (Kim et al., 2014). Taken together, these findings support a role for TDP-43 in translation, mediated at least in part through direct association with ribosomes via RACK1 protein.

### Local Dysregulation of Translation: Axons and Dendrites

#### RNP Granule Dynamics and Transport—A Role for TDP-43 in mRNA Localization

Neural plasticity during development and synaptic remodeling relies on the local synthesis of proteins in axons and dendrites (Holt et al., 2019). Within neurites, mRNAs are transported within RNP granules, which consist of mRNAs, ribosomes, translation factors and RNA binding proteins (Buffington et al., 2014; Glock et al., 2017). While mRNAs remain translationally inactive during transport within dendrites, they can be released from RNP granules in response to local activation and made available for translation at synaptic sites (reviewed in Akbalik and Schuman, 2014). Thus, RNA binding proteins play a critical role in RNP granule dynamics and, consequently, mRNA localization and translation (Kiebler and Bassell, 2006; Ayloo et al., 2017; Lim et al., 2017). Previous work has shown that TDP-43 is actively transported in motor neuron axons and mediates mRNA delivery to distal neuronal compartments, a mechanism that is impaired by ALS-linked mutations in TDP-43 (Fallini et al., 2012; Alami et al., 2014). Several reports indicate that TDP-43 colocalizes with the RNA binding proteins FMRP and Staufen1 in cultured neurons or *in vivo*, in Drosophila neurons, and cooperates with FMRP, an established translational regulator, to repress the translation of common mRNA targets (Wang et al., 2008; Majumder et al., 2012, 2016; Yu et al., 2012; Coyne et al., 2015).

The physical association between TDP-43, FMRP and Staufen in post-synaptic RNP granules was recently confirmed in primary mouse cortical neurons using super-resolution fluorescence microscopy (dSTORM) (Wong et al., 2021). To mechanistically investigate the potential co-regulation of RNP granule transport in dendrites via TDP-43, Staufen1, and FMRP, molecular beacons were used to characterize the dynamics of *Rac1* RNP granules in primary hippocampal neurons (Chu et al., 2019). These elegant experiments indicate that TDP-43 cooperates with FMRP and Staufen1 for the transport of a specific subset of dendritic mRNAs. Upon depletion of TDP-43, *Rac1* mRNA containing RNP granules remain stationary or exhibit brief bi-directional movement, indicating that TDP-43 is required for dendritic transport of *Rac1* transcripts (Chu et al., 2019). Taken together, these findings support a role for TDP-43 in efficient mRNA transport and localization in neurons under basal conditions and in response to activity.

#### Evidence for TDP-43 Mediated Local Translation

**FIGURE 2:**
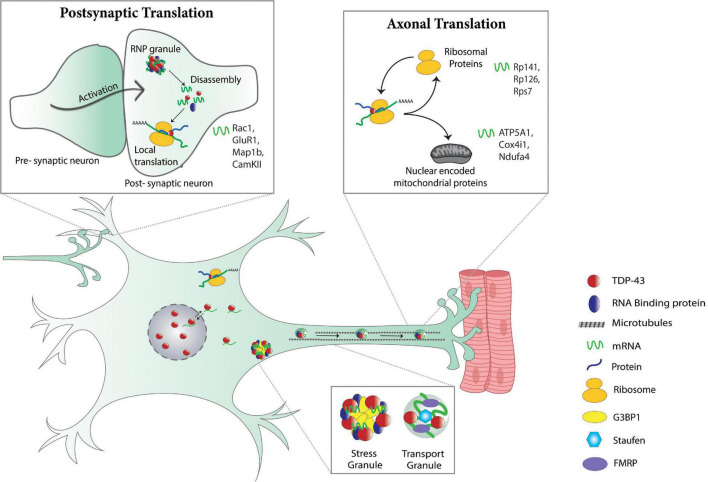
Physiological control of local translation by TDP-43. Post-synaptic translation (top left panel): in dendrites, activation of the post-synaptic neuron triggers the dissolution of dendritic RNP granules that in turn, causes the release of translationally silenced mRNAs making them available for translation. TDP-43 is a component of dendritic RNP granules and regulates dendritic translation of various mRNAs (*Rac1, GluR1, Map1b, CamKII)* (Wong et al., 2021). Stress and transport granules (bottom panel): TDP-43 associates with stress and RNA transport granules and is necessary for dendritic (Chu et al., 2019) and axonal transport (Alami et al., 2014) of mRNA-containing RNP granules. Axonal translation (top right panel): in axons, TDP-43 regulates local translation of mRNAs for ribosomal proteins (*Rp141, Rp126, Rps7*) and nuclear encoded mitochondrial proteins (*ATP5A1, Cox4i1, Ndufa4*) (Nagano et al., 2020; Altman et al., 2021).

In addition to its role in mRNA localization, TDP-43 was shown to be necessary for inhibition of translation within actively transporting RNP granules (Chu et al., 2019; see Figure 2). Indeed, depletion of TDP-43 by siRNA caused reduced entry of *Rac1* mRNA into dendritic spines and premature translation as evidenced by an elegant TRICK RNA reporter that allows real time visualization of mRNA transport and translation (Chu et al., 2019). Substantiating this conclusion are findings that neuronal activation in wild type neurons but not in neurons with TDP-43 proteinopathy induces the disassembly of TDP-43-containing RNP granules, which in turn causes the release of TDP-43 target mRNAs for subsequent local translation at the synapse, as evidenced by puromycin labeling and immunofluorescence of target mRNAs (Wong et al., 2021). Taken together, these results suggest that TDP-43 proteinopathy impairs postsynaptic local translation and RNA metabolism through perturbation of activity-dependent RNP granule dynamics (Wang et al., 2008; Yu et al., 2012; Wong et al., 2021).

Proper analysis of the spatiotemporal regulation of local translation in axons and at synaptic terminals requires the ability to experimentally manipulate them, independent from the soma. A recent report describes a novel platform for studying motor neuron axons and neuromuscular junctions by co-culturing primary motor neurons and muscles in microfluidic chambers, enabling fluidic separation and experimental manipulation of the motor neuron cell-bodies and axons, respectively (Altman et al., 2021). This setup allowed for compartment specific measurements of protein synthesis based on selective labeling with O-propargyl-puromycin (OPP) of nascent proteins in the motor neuron cell body, axon, or at the neuromuscular junction. Using the doxycycline (dox) TET-off system, transient accumulation of cytoplasmic TDP-43 (ΔNLS-TDP-43) was induced in axons of motor neurons, which resulted in the assembly of G3BP1-nucleated RNP condensates containing TDP-43. Formation of RNP condensates correlated with a reduction in local protein synthesis in axons and presynaptically at the NMJs, consistent with *in vivo* findings in ΔNLS-TDP-43 transgenic mice (Altman et al., 2021; Figure 2). Importantly, local protein synthesis recovered after dissolution of TDP-43 RNP condensates by a G3BP1 peptide (TAT-fused to residues 190–208 of G3BP1), suggesting that the reduction in local protein synthesis is a function of mRNA sequestration by TDP-43 RNP condensates. Clearance of TDP-43 from the cytoplasm by re-introducing doxycycline resulted in similar restoration of local protein synthesis and, remarkably, reinnervation of neuromuscular junctions, as evidenced by the co-expression of pre- and post-synaptic markers (Altman et al., 2021).

Additional evidence connecting mRNA transport and translation deficits was obtained from live imaging and microarray analyses of mouse cortical neurons depleted of TDP-43 (Nagano et al., 2020). RNAi knock-down of TDP-43 caused downregulation of a particular subset of ribosomal protein (RP) mRNAs in neurites but not in the cell-body, as shown by microarray analysis. To track the transport of RP mRNA by TDP-43 in living cells, fluorescence microscopy was conducted on cultured cortical neurons simultaneously expressing mCherry TDP-43 and mRNAs labeled using the MS2 tagging system (Bertrand et al., 1998). Loss of TDP-43 results in decreased axonal transport and subsequent local translation of RP mRNAs, along with diminished translational activity in axons. Further investigation by *in situ* hybridization of RP mRNAs, both with and without 5′UTRs, and TDP-43 immunostaining revealed that the 5′UTR is necessary for TDP-43-mediated axonal transport of RP mRNAs (Nagano et al., 2020). Taken together, these results show that TDP-43 proteinopathy impairs axonal translation with direct consequences on neuromuscular junction integrity and ribosomal protein expression, which in turn can cause more general alterations in local translation (Nagano et al., 2020; Altman et al., 2021).

### Specific Candidate Targets as Mediators of TDP-43 Toxicity

Although TDP-43 appears to play a critical role in modulating RNP granule dynamics and mRNA localization, identifying specific translational targets of TDP-43 remains paramount in the development of novel therapeutics. Early reports identified *Rac1* and *futsch*/*Map1b*, key regulators of neural plasticity, as specific mRNA targets of TDP-43 (Majumder et al., 2012; Coyne et al., 2014). Rac1 is a known positive regulator of AMPAR-mediated spinogenesis and is shown to be upregulated upon depletion of TDP-43 in mouse hippocampal neurons (Majumder et al., 2012). Consistent with these findings, TDP-43 has been shown to cooperate with FMRP to co-repress the translation initiation of *Rac1*, *GluR1*, and *Map1b* in dendrites (Majumder et al., 2016). In the context of TDP-43 proteinopathy modeled by TDP-43 overexpression, neuronal activation-induced dissolution of post-synaptic RNPs containing *Map1b*, *GluR1*, and *CamkII* is impaired and accompanied by reduced translation as evidenced by ribopuromycylation assays (Wong et al., 2021). In Drosophila, overexpression of TDP-43 (wild type or the disease associated variant G298S) in motor neurons represses the translation of *futsch*, as evidenced by a reduction in Futsch protein expression at the NMJ and a shift in *futsch* mRNA from actively translating polysome fractions to non-translating RNPs (Coyne et al., 2014). Dysregulation of *futsch* mRNA reduces microtubule stability at the neuromuscular junction, which is mitigated by restoring *futsch* levels using overexpression in the context of TDP-43 proteinopathy (Coyne et al., 2014). Another mRNA target with functional consequences on synaptic physiology is *hsc70-4* mRNA (Coyne et al., 2017). More specifically, TDP-43 impairs the translation of *hsc70-4* mRNA due to its sequestration by mutant TDP-43^G298S^, as evidenced by soluble vs. insoluble and polysome fractionations (Coyne et al., 2017). Upon restoration of *hsc70-4* levels via overexpression, synaptic vesicle endocytosis measured using FM1-43 dye uptake is rescued, suggesting that dysregulation of *hsc70-4* translation mediates TDP-43 toxicity at the neuromuscular synapse (Coyne et al., 2017). To identify specific mRNAs transported by TDP-43, *in vitro* systematic evolution of ligands by exponential enrichment (SELEX) demonstrated that RNAs bound by TDP-43 contained G-quadruplexes and were transported to distal neurites along with TDP-43 for local translation, consistent with G-quadruplexes being novel structural motifs within RNAs associated with TDP-43 (Ishiguro et al., 2016).

Additional translational targets of TDP-43 have recently been reported. In aged mice expressing mutant TDP-43^A315T^, TRAP and microarray analyses revealed seven genes that are differentially expressed. Of these, four candidates (*Ddx58*, *Ccl4*, *Prickle4*, and *Mthfsd*) were confirmed by immunofluorescence in mouse spinal cord motor neurons. Immunohistochemistry experiments in patient derived spinal cords confirmed that DDX58 and MTHFSD, both of which are RNA binding proteins, are differentially expressed in ALS compared to controls (MacNair et al., 2016). Another study characterized changes to the motor neuron translatome in TDP-43^A315T^ transgenic mice, specifically at the onset of motor symptoms (Marques et al., 2020). By comparing transgenic mice with wild type littermates and asymptomatic mice hemizygous for the wild type hTDP-43 transgene (*Chat* bacTRAP; *hTDP-43*^*WT*^), translational changes specifically associated with disease could be distinguished. Candidate mRNAs *Syngr4* and *Plekhb1* were up- and downregulated, respectively, at the transition from asymptomatic to early symptomatic motor dysfunction, as identified by TRAP and RNA sequencing approaches in spinal cord motor neurons (Marques et al., 2020). Validation experiments confirmed that SYNGR4 and PLEKHB1 protein levels were dysregulated as predicted. Interestingly, these targets were altered in two different mouse models of TDP-43 proteinopathy (TDP-43^A315T^ and TDP-43^Q331K^), suggesting that they may play critical role in the progression of mutant TDP-43 driven ALS (Marques et al., 2020).

Moreover, overexpression of TDP-43 (wild type and A315T) in motor neuron-like cells and in primary cortical neurons enhanced the translation of *Camta1* and *Mig12* mRNAs through 5′UTR binding. Conversely, translation of *Dennd4a* was enhanced specifically by mutant TDP-43^A315T^ through the 3′UTR (Neelagandan et al., 2019). Importantly, independent studies in cultured ALS motor neurons and an *in vivo* murine model of Parkinson’s disease identified CAMTA1 and DENND4A as “master regulators” of transcriptional programs in neurodegenerative disease (Brichta et al., 2015; Ikiz et al., 2015).

A more recent study using RNA immunoprecipitation assays, TRAP, and bioinformatics analyses, reported the glypican Dally like protein (Dlp) as a novel TDP-43 candidate target, based on the enrichment of *dlp* mRNA in TDP-43 complexes and depletion from ribosomes in the context of TDP-43 proteinopathy (Lehmkuhl et al., 2021). This study further shows that surprisingly, while Dlp expression in synaptic terminals at the neuromuscular junction is significantly reduced, Dlp protein accumulates in puncta within the ventral cord neuropil suggesting that a combination of translation and transport defects are at play, and that these effects may be compartment specific. Interestingly, TDP-43 knock-down by RNAi (TBPH^RNAi^) was sufficient to deplete Dlp from the NMJ, but not induce significant ventral cord neuropil puncta, highlighting that TDP-43 nuclear depletion and cytoplasmic accumulation have distinct contributions to disease pathomechanism. Restoring Dlp by overexpression, in motor neurons specifically, restores Dlp expression at the synaptic terminal and mitigates TDP-43-dependent locomotor deficits. Importantly, these findings align with ALS patient data indicating that GPC6 protein, a human homolog of Dlp, accumulates in puncta within the spinal cord, mimicking the Dlp accumulations in the Drosophila ventral cord neuropil (Lehmkuhl et al., 2021). Additional targets identified in Drosophila using TRAP include metabolic pathways (e.g., pentose phosphate, nuclear encoded components electron transport chain components, oxidative stress). Of note, overexpressing glucose 6 phosphate dehydrogenase (G6PD), which is predicted to be downtranslated in the context of TDP-43^G298S^, mitigates locomotor deficits in Drosophila models and highlights a role for metabolic rewiring in degenerating motor neurons (Lehmkuhl et al., 2021).

Interestingly, several recent studies have identified mRNA targets of TDP-43 that encode distinct populations of proteins, such as ribosomal proteins (RPs), mitochondrial proteins, and translation factors (Nagano et al., 2020; Altman et al., 2021; Gao et al., 2021; Lehmkuhl et al., 2021). For instance, cytoplasmic TDP-43 caused a specific decrease in nuclear-encoded mitochondrial proteins, particularly ATP5A1, Cox4i1, and Ndufa4 (Altman et al., 2021). Notably, mRNA levels of these three proteins were unchanged and even moderately increased in TDP-43ΔNLS samples, suggesting TDP-43 accumulation in axons directly impairs local translation of nuclear encoded mitochondrial proteins (Altman et al., 2021). TDP-43 depletion has also been shown to reduce the number and functionality of mitochondria in MN axons, and subsequent treatment with nicotinamide (NAM), a precursor for nicotinamide adenine dinucleotide (NAD +), rescues axon growth defects and capacity for protein synthesis (Briese et al., 2020). In agreement with these findings, depletion of TDP-43 in cortical neurons causes a specific downregulation of ribosomal protein mRNAs in neurites that coincides with axonal extension defects, which are rescued upon overexpression of RP mRNAs Rp141, Rp126, or Rps7 (Nagano et al., 2020). In addition to translational changes in mitochondrial and ribosomal protein mRNA, several translation factors, namely PABPC4, PABPC1, RPS6, EEF1A1, and RPL7, have been shown to co-precipitate with wild type TDP-43 and are enriched in TDPΔCR mutant mice (Gao et al., 2021). Taken together, these findings highlight a role for TDP-43 in regulating various cellular pathways including synaptic metabolism, cellular energetics, and cytoplasmic translation (Marques et al., 2020; Nagano et al., 2020; Altman et al., 2021; Lehmkuhl et al., 2021).

## Discussion

Growing evidence of alterations to the translatome in TDP-43 proteinopathies supports a multifaceted role for TDP-43 involvement in translation, highlighting both physiological and pathogenic functions. TDP-43 knock-down studies demonstrate that TDP-43 is necessary for the RNP-mediated transport of mRNAs into axons (Alami et al., 2014; Nagano et al., 2020) and dendrites (Chu et al., 2019), and that loss of TDP-43-mediated transport leads to a reduction in local translation. Underscoring the importance of mRNA transport in fueling local protein synthesis, these findings suggest that TDP-43 might dysregulate translation, in part, through indirect mechanisms involving the supply of mRNA to distal neurites. Indeed, additional studies have recently shown that cytoplasmic accumulation of TDP-43 disrupts RNP granule dynamics, impairing local translation in dendrites (Wong et al., 2021) and axons (Altman et al., 2021). Consistent with the ribostasis hypothesis (Ramaswami et al., 2013), cytoplasmic TDP-43 appears to inhibit translation by sequestering mRNAs in aberrant RNP granules, suggesting that TDP-43 intimately attenuates mRNA accessibility in neurites and thus alterations in TDP-43 structure or localization could destabilize the chain of events preceding local translation.

Although TDP-43 dependent alterations in the global translatome are well documented (Fiesel et al., 2012; MacNair et al., 2016; Russo et al., 2017; Neelagandan et al., 2019; Charif et al., 2020; Lehmkuhl et al., 2021), and TDP-43 co-fractionates with actively translating ribosomes (Coyne et al., 2015; Neelagandan et al., 2019), evidence for its direct association with ribosomes has only recently been uncovered (Russo et al., 2017) and requires further investigation, perhaps using more powerful structural techniques such as cryo-EM. Complicating the understanding of TDP-43’s role in translation is its global effect on cytoplasmic translation itself (Lehmkuhl et al., 2021). Mechanistically, this could occur via direct transport and translation regulation of ribosome protein mRNAs, specifically via the 5′UTR, as recently demonstrated (Nagano et al., 2020).

**FIGURE 3:**
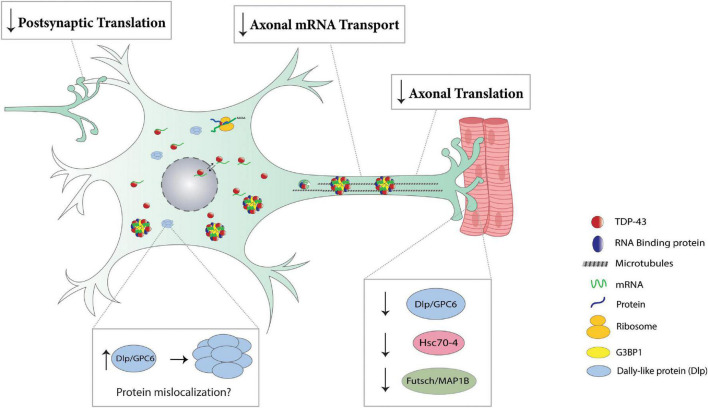
Translation impairments in TDP-43 proteinopathies. Post-synaptically, TDP-43 proteinopathy prevents the dissolution of dendritic RNP granules (Wong et al., 2021), thereby repressing the local translation of key mediators of synaptic plasticity (*Rac1, GluR1, Map1b, CamKII)*. Cytoplasmic accumulation of TDP-43 in axons results in the formation of RNP condensates and causes a reduction in local translation of various mRNAs, including ribosomal proteins (*Rp141, Rp126, Rps7)* and nuclear encoded mitochondrial proteins (*ATP5A1, Cox4i1, Ndufa4*) (Nagano et al., 2020; Altman et al., 2021). TDP-43 proteinopathy reduces axonal mRNA transport as evidenced by a reduction in axonal levels of *futsch* mRNA at the Drosophila NMJ (Coyne et al., 2015). In Drosophila, TDP-43 proteinopathy represses the translation of *dlp*, *futsch/Map1b*, and *hsc70-4* causing a reduction in respective protein expression at the NMJ (bottom right panel) (Coyne et al., 2015, 2017; Lehmkuhl et al., 2021). TDP-43 proteinopathy causes Dlp/GPC6 protein to accumulate in puncta at the ventral cord neuropil in Drosophila and ALS spinal cords, respectively (bottom left panel) (Lehmkuhl et al., 2021).

Regardless of mechanism, it is clear that TDP-43 accumulation in the cytoplasm is driving translation dysregulation (see Figure 3). The identification of specific targets is critical for understanding the mechanisms underlying TDP-43 dependent proteinopathies and has already uncovered common alterations across models, such as cellular metabolism, synaptic function and cytoplasmic translation (Coyne et al., 2014, 2017; Majumder et al., 2016; Chu et al., 2019; Nagano et al., 2020; Altman et al., 2021; Lehmkuhl et al., 2021). However, developing therapeutic strategies that mitigate neurodegeneration one target at a time does not seem like a practical approach as prioritizing targets is difficult and would require a combination strategy that simultaneously affects multiple pathways, one for each target. A possible solution to this challenge is the restoration of TDP-43 localization to the nucleus, which was shown to cause the reinnervation of neuromuscular junctions (Altman et al., 2021). That said, strategies to enhance translation locally at synapses could also offer mitigating approaches to TDP-43 dependent neurodegeneration.

Questions remain on the potential connections between TDP-43’s role in transcription, splicing, RNA stability and translation that could help identify additional strategies for restoring more downstream translation deficits. A significant caveat is the limited knowledge regarding the physiological role of TDP-43 in RNA processing within the cytoplasm. The discovery of the cytoplasmically localized sTDP-43 (Weskamp et al., 2020), the increased availability of endogenously tagged TDP-43 models, and higher resolution imaging technologies, may help overcome this barrier and lead to a better understanding of the mechanistic differences between healthy and degenerating neurons that could in turn uncover novel therapeutic strategies for ALS and related neurodegenerative disorders.

## Author Contributions

RTB, NPM, and DCZ wrote the manuscript. SL, RTB, and NPM made the figures. All authors contributed to the article and approved the submitted version.

## Conflict of Interest

The authors declare that the research was conducted in the absence of any commercial or financial relationships that could be construed as a potential conflict of interest.

## Publisher’s Note

All claims expressed in this article are solely those of the authors and do not necessarily represent those of their affiliated organizations, or those of the publisher, the editors and the reviewers. Any product that may be evaluated in this article, or claim that may be made by its manufacturer, is not guaranteed or endorsed by the publisher.
